# Low‐intensity exercise stimulates bioenergetics and increases fat oxidation in mitochondria of blood mononuclear cells from sedentary adults

**DOI:** 10.14814/phy2.14489

**Published:** 2020-06-19

**Authors:** Edgars Liepinsh, Elina Makarova, Liga Plakane, Ilze Konrade, Kaspars Liepins, Melita Videja, Eduards Sevostjanovs, Solveiga Grinberga, Marina Makrecka‐Kuka, Maija Dambrova

**Affiliations:** ^1^ Latvian Institute of Organic Synthesis Riga Latvia; ^2^ University of Latvia Riga Latvia; ^3^ Riga Stradins University Riga Latvia

**Keywords:** exercise, fat metabolism, lipolysis, obesity, sedentary adults

## Abstract

**Aim:**

Exercise training induces adaptations in muscle and other tissue mitochondrial metabolism, dynamics, and oxidative phosphorylation capacity. Mitochondrial fatty acid oxidation was shown to be pivotal for the anti‐inflammatory status of immune cells. We hypothesize that exercise training can exert effects influence mitochondrial fatty acid metabolism in peripheral blood mononuclear cells (PBMCs). The aim was to investigate the effect of exercise on the fatty acid oxidation‐dependent respiration in PBMCs.

**Design:**

Twelve fasted or fed volunteers first performed incremental‐load exercise tests to exhaustion on a cycle ergometer to determine the optimal workload ensuring maximal health benefits in volunteers with a sedentary lifestyle. In addition, the same volunteers performed 60 min of low‐intensity constant‐load exercise.

**Results:**

In the incremental‐load exercise, the maximal whole‐body fat oxidation rate measured by indirect calorimetry was reached at the fasted state already at a 50 W workload. At the 75–175 W workloads, the contribution of fat oxidation significantly decreased to only 11%, the heart rate increased to 185 BPM, and the study participants reached exhaustion. These results show that low‐intensity exercise (50W) is optimal for maximal whole‐body fat utilization. After low‐intensity exercise, the ROUTINE mitochondrial respiration, as well as fatty acid oxidation‐dependent respiration in PBMCs at LEAK and OXPHOS states, were significantly increased by 31%, 65%, and 76%, respectively. In addition, during 60 min of low‐intensity (50W) exercise, a 2‐fold higher lipolysis rate was observed and 13.5 ± 0.9 g of fat was metabolized, which was 57% more than the amount of fat that was metabolized during the incremental‐load exercise.

**Conclusions:**

In individuals with a sedentary lifestyle participating in a bicycle ergometry exercise program, maximal lipolysis and whole‐body fat oxidation rate is reached in a fasted state during low‐intensity exercise. For the first time, it was demonstrated that low‐intensity exercise improves bioenergetics and increases fatty acid oxidation in PBMCs and may contribute to the anti‐inflammatory phenotype.

## 1 INTRODUCTION

Obesity is a major public health problem that affects over 600 million adults worldwide, and physical activity is the most important intervention not only for the weight management, but also for the prevention of metabolic and cardiovascular diseases (Golbidi & Laher, 2012; MacLeod, Terada, Chahal, & Boule, 2013; Malin et al., 2013; Sacre et al., 2014). Adaptive response to training is primarily translated into increased muscle endurance, enhanced vascular functions, and metabolism oxidative capacity. Multiple findings indicate that the benefit of exercise extend beyond the musculoskeletal system, cardiovascular, and metabolic diseases (Luan et al., 2019). Training induces improvements in mitochondrial structure and function and results in increased quantity as well as a profound impact on mitochondrial dynamics—fusion and fission (Drake, Wilson, & Yan, 2016; Merry & Ristow, 2016). Most of the findings related to mitochondrial bioenergetics are clearly evidenced in skeletal muscle, however, it is poorly studied in other cell types. Results regarding exercise effects on mitochondrial function in PBMCs are inconclusive. Maximal respiration and spare respiratory capacity of PBMCs mitochondria correlated with gait speed in older adults (Tyrrell et al., 2015). Training increased the mitochondrial biogenesis and improved the antioxidant capacity of mitochondria in well‐trained individual PBMCs (Busquets‐Cortes et al., 2017). In a recent study, the mitochondrial function of PBMCs did not reflect interval training‐induced changes in muscle mitochondria of young healthy men (Hedges et al., 2019). Considering the variability of exercise effects on different diseases and conditions, the recommended type, intensity, and frequency of exercise also should be clarified. We hypothesize that exercise training can affect cell functionality and, thereby, can exert some effects on mitochondrial energy metabolism that have not been described in PBMCs.

Carbohydrates and lipids are the main energy sources during exercise. The amount of intracellular triglycerides is comparably limited, and during prolonged exercise, fatty acids are obtained via the lipolysis of circulating lipids and from free fatty acids in adipose tissue (Jeppesen & Kiens, 2012). During exercise, the utilization of carbohydrates and fat significantly increases; however, these changes are not consistent among various exercise intensities. The maximal energy contribution of lipid oxidation occurs primarily at exercise intensities of approximately 50% of the VO_2_max and is also complementary to carbohydrate oxidation (van Loon, Greenhaff, Constantin‐Teodosiu, Saris, & Wagenmakers, 2001; Venables, Achten, & Jeukendrup, 2005). At higher exercise intensities, fat oxidation is limited because of restricted fatty acid flux and the subsequent mitochondrial metabolism (Jeppesen & Kiens, 2012). Increases in glucose uptake, glycolysis, and glucose oxidation are not limited, and at exercise intensities over 50% of the VO_2_max, carbohydrates become the predominant energy source and the fat oxidation rate begins to decline (Achten & Jeukendrup, 2003; Venables et al., 2005). Training status, exercise intensity and duration, and nutritional status have all been shown to affect the maximal fat oxidation capacity which is an important characteristic of mitochondria metabolism. Therefore, the purpose of the present study was to determine the optimal conditions to increase fat oxidation rates in the context of different exercise intensities and nutritional statuses.

Mitochondrial fatty acid oxidation was shown to be pivotal for immune cells anti‐inflammatory status (Michalek et al., 2011; Vats et al., 2006; Weinberg, Sena, & Chandel, 2015). Various aspects of mitochondrial dysfunction have previously been shown as contributing to inflammatory diseases (Li et al., 2015; Makrecka‐Kuka et al., 2019; Weiss et al., 2019). Recent findings indicate on a metabolic basis for the inflammatory response and could suggest mitochondrial functionality as a new therapeutic target for controlling the immune response and delaying the onset of diseases (Agarwal et al., 2019). Thus metabolic adaptation in the immune cells is necessary to modulate immune cell functions as they are intricately coupled with intracellular metabolism (Fuller, Summers, & Valentine, 2017). Considering the beneficial effects of exercise on mitochondria functionality, this could be a promising possibility to modulate immune cell metabolism and functions. A better understanding of the factors regulating fat oxidation is important for the development of interventions for obesity. We aimed to find an optimal workload and then characterize the metabolic state of human PBMCs after exposure to exercise. We hypothesize that low‐intensity exercise in a fasted state would be optimal for fat oxidation in healthy volunteers with a sedentary lifestyle.

## 2 MATERIALS AND METHODS

### 2.1 Volunteers

Twelve volunteers participated in a crossover study, which was approved by the local Riga Stradins University Ethics Committee, Latvia (ClinicalTrials.gov identifier: NCT03864679). All volunteers had sedentary lifestyles and were healthy as assessed by a general health questionnaire; no volunteers were accepted into the study if they had diabetes or were medically treated for any cardiovascular diseases. The physical activity levels of the participants were determined by the World Health Organization‐endorsed Global Physical Activity Questionnaire (GPAQ) using the GPAQ Analysis Guide. All volunteers were informed of the purpose and nature of the study.

According to a self‐evaluation, the average time spent being sedentary was 462 ± 37 min/day (45% of waking hours). The self‐reported time spent participating in moderate‐to‐vigorous physical activity (MVPA) of the adult volunteers was 227 ± 51 min/week. The average MVPA, moderate physical activity (MPA) and vigorous physical activity (VPA) of the participants were 1,030, 783, and 247 the metabolic equivalent of task MET min/week, respectively.

### 2.2 Experimental design

**FIGURE 1:**
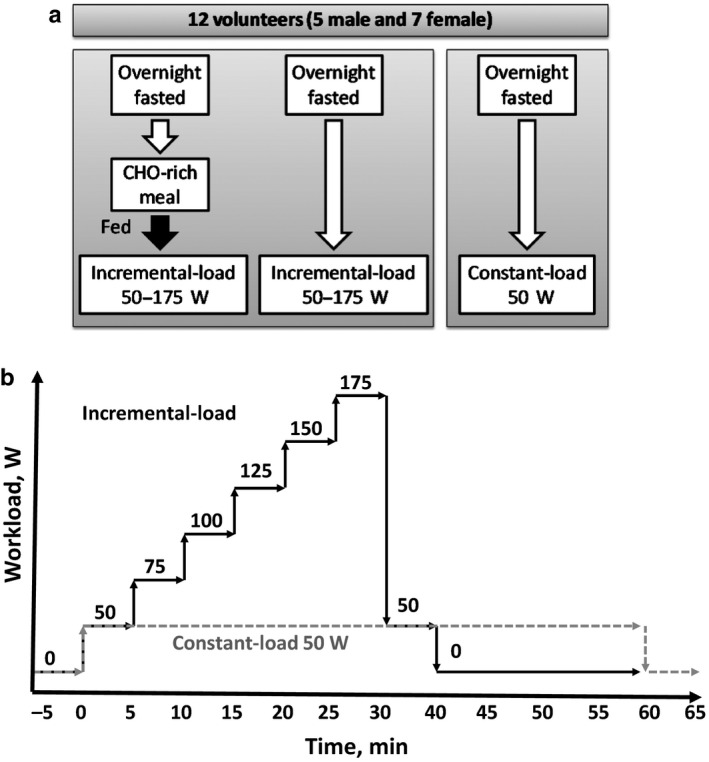
Experimental design related to nutritional status (a) and workload (b) in the context of an incremental‐ or low‐intesity constant workload exercise programme

The main physical characteristics of the 12 volunteers are shown in Table 1. Each subject performed three laboratory‐based trials, which were separated by at least 14 days. The experimental design in the context of exercise intensity versus time is plotted in Figure 1. At the beginning we compared muscular metabolism in the same individuals at the fed and fasted states, therefore initially, volunteers performed cross‐over incremental‐load single‐exercise bout tests to exhaustion on a bicycle ergometer (Ergoline, ergoselect 100/200 GmbH, Bitz, Germany) in the fasted and postprandial state in a random order (Figure 1a). Each volunteer performed incremental‐load workout at both fed and fasted states. To standardize the fed condition, all participants arrived in a sports laboratory after overnight fasting and received a standardized carbohydrate‐rich meal. The experimental design in the context of exercise intensity versus time is plotted in Figure 1b. Based on obtained data in the preliminary incremental‐load experiments, the lowest 50W workload at the fasted state was selected as optimal for maximal whole‐body fat utilization. This hypothesis was then tested in a proof of concept constant‐load exercise experiment at the 50 W workload. Whole body Fat and carbohydrate oxidation was determined by indirect calorimetry and plotted as a function of time. The rate of extra‐muscular fatty acid oxidation‐dependent mitochondrial respiration was measured in permeabilized PBMCs.

**TABLE 1:** Physiological characteristics of the volunteers

Age, years	Gender, M/F	Height, cm	Weight, kg	BMI, kg/m^2^	VO_2_max, ml/min/kg
36.2 ± 7.3	5/7	175.9 ± 12.7	78.1 ± 14.3	25.1 ± 2.5	33.3 ± 1.3

Note:

Values are the mean ± *SEM* of the 12 volunteers.

Subjects arrived at the laboratory (Laboratory of Human and Animal Physiology, University of Latvia) after an overnight fast. They had all been instructed to avoid strenuous exercise for the previous 24 hr. To study exercise in the postprandial state, 30 min before exercise, all subjects consumed a standardized meal (343 kcal of total energy) consisting of carbohydrates (241 kcal, sugar 170 kcal), fat (46 kcal), and protein (56 kcal).

The resting state and recovery measurements were performed 5 min before and 15 min after exercise, respectively. Each volunteer was asked to maintain a constant cycle ergometer cadence at 50 rpm during the entire exercise bout. The volunteers started exercising at a workload of 50 W. During the incremental‐load exercise, the workload was increased by 25 W every 5 min until exhaustion was reached. In the constant‐load exercise, the workload was 50 W for 60 min. Breath‐by‐breath measurements were taken throughout exercise by using an automated gas analysis system (Masterscreen CPX CareFusion, San Diego, USA). Heart rate was recorded continuously using a 4‐lead ECG (Tango+, SunTech Medical, Morrisville, USA).

Oxygen uptake (VO_2_) and carbon dioxide production (VCO_2_) were averaged over the last 2 min of each exercise stage. For each stage, fat and carbohydrate oxidation and energy expenditure were calculated by using stoichiometric equations that were built into the software with the assumption that the urinary nitrogen excretion rate was negligible. Substrate oxidation rates were then plotted as a function of time. Maximal VO_2_ was determined by a submaximal prediction test based on the linear relationship between HR and VO_2_ when a subject was exercising at submaximal levels; the heart rate was used to predict the maximal performance either by extrapolating to HRmax or by using HR at a known power output. The subject's heart rates at each workload were plotted, and the line of best fit was determined: the point on the line that coincided with the estimated maximal heart rate provided an estimate of VO_2_max (Evans, Ferrar, Smith, Parfitt, & Eston, 2015). The maximal heart rate was determined using the equation for estimating age‐predicted HRmax: 208–0.7 x age (Tanaka, Monahan, & Seals, 2001).

### 2.3 Glucose, lactate, fatty acid measurements

For biochemical measurements before and after exercise, blood samples were collected from the vein in heparin‐containing tubes. To obtain plasma, the samples were centrifuged at 1,000× *g* for 10 min at 4°C. All samples were stored at −80°C until analysis. The plasma glucose concentrations were determined using a kit from Instrumentation Laboratory. The lactate level was measured in the samples using an enzymatic kit from Roche Diagnostics (Mannheim, Germany). The concentration of free fatty acids was measured using a commercially available enzymatic kit from Wako (Neuss, Germany).

### 2.4 Measurement of acylcarnitine levels by UPLC/MS/MS

The concentrations of acylcarnitines in the plasma and PBMC samples were determined with a UPLC MS/MS method using a Waters ACQUITY liquid chromatography system and a Waters Quattro Micro or Waters Xevo TQ‐S mass spectrometer, as previously described (Liepinsh et al., 2017).

### 2.5 Peripheral blood mononuclear cells (PBMCs) respirometry

PBMCs were isolated before and after constant‐load 50W workout as described previously (Karabatsiakis et al., 2014) and respirometry was performed using Oxygraph‐2k (O2k; OROBOROS INSTRUMENTS, Innsbruck, Austria). Peripheral blood (15 ml) was collected from the vein in heparin‐containing tubes. A Ficoll‐PaqueTM PLUS (density gradient 1.077 g/ml) was used to isolate PBMCs. Briefly, whole blood was diluted with PBS (1:1 ratio) and layered on the top of density gradient media. PBMCs were separated by centrifugation at 1,000× *g* 10 min with no brakes. Then cells were washed twice with PBS. Isolated PBMCs were frozen gradually decreasing temperature and stored at − 80°C in standard cryoprotective freezing medium (DMSO: Sigma–Aldrich, St. Louis, MO, USA; fetal calf serum: Sigma–Aldrich; dilution: 1:10) until analysis. Frozen PBMCs were thawed, counted, and resuspended in mitochondria respiration media for further mitochondrial functionality assessment or acylcarnitine profile analysis.

The PBMC mitochondrial respiration was performed in 2 × 10^6^ PBMCs per ml at 37°C using an Oxygraph‐2k (O2k; OROBOROS INSTRUMENTS, Innsbruck, Austria) in MiR05 media (110 mM sucrose, 60 mM K‐lactobionate, 0.5 mM EGTA, 3 mM MgCl_2_, 20 mM taurine, 10 mM KH_2_PO_4_, 20 mM HEPES, pH 7.1, 0.1% BSA essentially free of fatty acids). Firstly, ROUTINE state that characterizes the respiration of intact cells in the physiological coupling state (before the addition of any substrates or inhibitors) was assessed in intact PBMCs. Then cells were permeabilized using digitonin (5 µg/ml), and the fatty acid oxidation‐dependent mitochondrial respiration in permeabilized PBMCs was measured using palmitoylcarnitine (10 µM), carnitine (100 µM) and malate (0.1 mM) as substrates for LEAK state, then ADP (5 mM) was added to evaluate OXPHOS state. In addition, complex IV‐linked respiration was measured using ascorbate (2 mM) and N,N,N′,N′‐tetramethyl‐p‐phenylenediamine dihydrochloride (TMPD, 0.5 mM) as substrates. The chemical background oxygen consumption induced by the auto‐oxidation of TMPD and ascorbate reactions was assessed after the inhibition of complex IV by sodium azide (100 mM).

### 2.6 Statistical analysis and exclusion criteria

The values are represented as the mean ± standard error of the mean (*SEM*). After assessing the normality of the data, statistically significant differences in total energy expenditure and substrate utilization rate between different exercise intensities and nutritional status were identified by using one‐way repeated measures ANOVA. If multiple comparisons were necessary, two‐way repeated measures ANOVAs (time x trial) were used to identify differences between trials. A paired *t* test was used to compare the differences in heart rate and mitochondrial functionality in PBMCs at baseline and after 50 W constant‐load exercise. The differences were considered significant when *p* < .05. The data were analyzed using GraphPad Prism statistical software (GraphPad Inc.).

PBMC samples from three volunteers were excluded from the mitochondrial respiration assay and acylcarnitine profile analysis since an insufficient for analysis number of cells was isolated.

## 3 RESULTS

**FIGURE 2:**
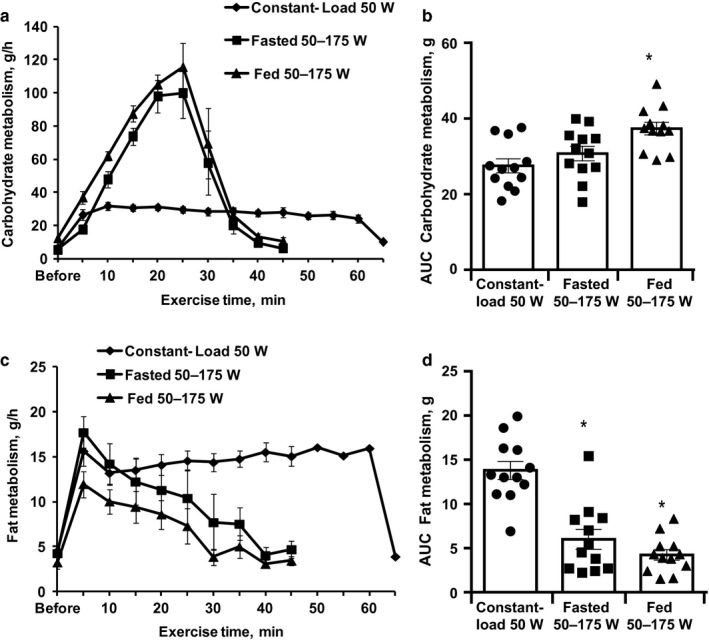
Carbohydrate metabolism rate during incremental‐load and constant‐load exercise at each time point (a) and the total carbohydrate oxidation rate during the experiment (b). Fat metabolism rate during incremental‐load and constant‐load exercise at each time point (c) and total fat oxidation during the experiment (d). Values are the mean ± *SEM* from 12 volunteers. *Significantly different from constant‐load exercise (one‐way repeated measures ANOVA, Tukey's multiple comparisons test, *p* < .05)

Initially, volunteers performed cross‐over incremental‐load single‐exercise bout tests to determine optimal workload. The carbohydrate oxidation rates in the resting state in fasted and fed subjects were 6.2 ± 1.1 and 12 ± 2.1 g/hr, respectively (Figure 2a). The increase in carbohydrate oxidation in the incremental‐load and constant‐load exercise experiments was similar to the increase in total energy expenditure. During the incremental‐load exercise, the carbohydrate oxidation rate gradually and significantly increased depending on the workload, and at higher workloads, it reached 100 ± 15 g/hour in the fasted state and 116 ± 15 g/hour in the fed state (Figure 2a). Thus, in comparison to the carbohydrate utilization in the resting state, carbohydrate utilization during the highest workload exercise in the fasted and fed states was 19‐ and 9.4‐fold higher, respectively. In the constant‐load experiment, carbohydrate oxidation significantly increased 5‐fold (30 ± 2.3 g/hr) at the beginning of the experiment and slightly decreased to 25 ± 2.3 g/hr at the end of the 60 min exercise experiment (Figure 2b). The total carbohydrate oxidation during the incremental‐load exercise at the fed state was significantly higher than that in the fasted state experiments.

The fat oxidation rates in the resting state in the fasted and fed subjects were 4.4 ± 0.6 and 3.3 ± 0.7 g/hr, respectively (Figure 2c). The increase in fat oxidation in the constant‐load experiment was similar to the increase in total energy expenditure, while in the incremental‐load experiment, fat oxidation at the lowest workload significantly increased but then started to decrease (Figure 2d). During incremental‐load exercise at 50 W, the fat oxidation rate reached a maximum of 17.7 ± 1.8 g/hr in the fasted state. In fed subjects, the corresponding maximum fat oxidation rate was 12 ± 1.6 g/hr (Figure 2c), thus it was significantly lower by 33% than in the fasted state.

Compared to the relative fat utilization in the resting state, the relative fat utilization during the lowest workload in the fasted and fed states significantly increased by approximately 4‐fold. In the incremental‐load experiment, a further increase in workload above 50 W induced a gradual decrease in fat oxidation. This decrease was observed in both the fed and fasted states. In contrast, in the constant‐load experiment, the fat oxidation rate was stable during the 60 min of exercise (Figure 2c). During the 60 min of the constant‐load experiment, the total fat oxidation was 13.8 ± 1.0 g (fasted state), while during the incremental‐load experiment, the fat utilization in the fed and fasted states was significantly lower ‐ 6.0 ± 1.1 and 4.2 ± 0.6 g, respectively (Figure 2d). Thus, the energy demand during high‐load exercise is mostly supplied by carbohydrate oxidation, and high workloads are not appropriate to stimulate fat oxidation.

**FIGURE 3:**
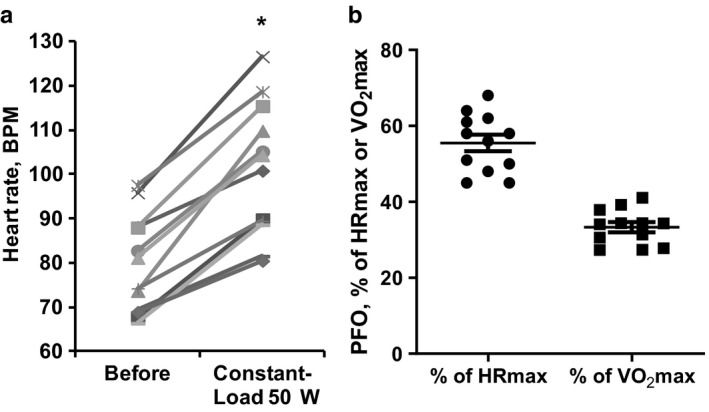
Heart rate of each individual before and during 50 W constant‐load exercise (a) and relative heart rate and oxygen uptake at peak fat oxidation (PFO) expressed as the percentage of the maximal heart rate (HRmax) and oxygen uptake (VO_2_max; b). Values are the mean ± *SEM* from 12 volunteers. *Significantly different from heart rate at baseline (paired *t* test, *p* < .05)

The heart rate at the resting state was variable between study subjects and ranged from 66 to 98 beats per minute (BPM) (Figure 3a). During exercise, in response to incremental workloads, the heart rate gradually increased, reaching up to 180 BMP at higher workloads (data not shown). In the incremental‐ and constant‐load tests, a 50 W workload induced a significant increase in heart rate from 13 to 36 BPM with an average of 21.4 BPM. In the constant‐load experiment, the heart rate remained stable during the 60 min of exercise). Thus, a heart rate increase of 20–25 BPM corresponds to the optimal fat oxidation level. The average maximal heart rate (HRmax) of the study subjects was 182 ± 1.5 BPM, while the maximal oxygen uptake (VO_2_max) was 33 ± 1.3 ml min^−1^ kg^−1^. In incremental‐ and constant‐load tests at a 50 W workload, the relative heart rate and oxygen uptake were 55.5 ± 2.2 and 36.0 ± 1.8% of the maximal, respectively (Figure 3b).

**FIGURE 4:**
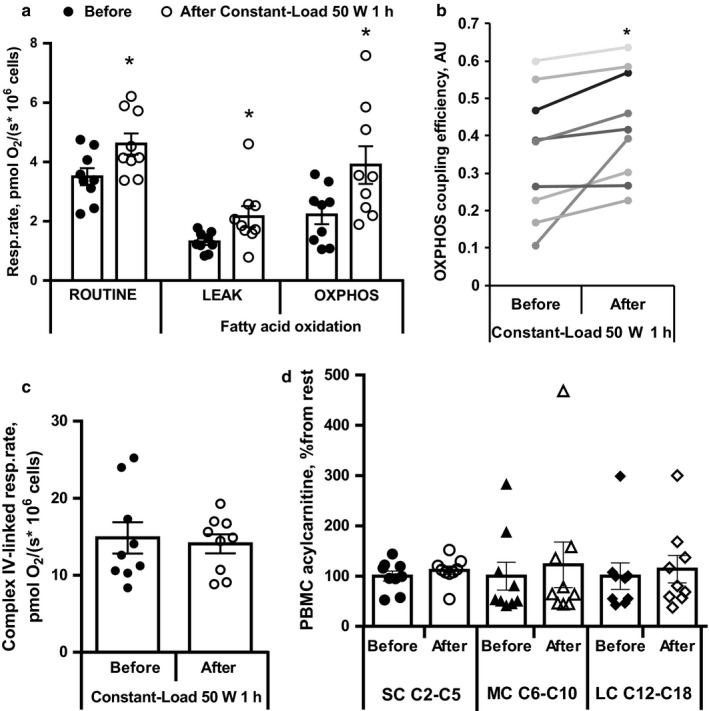
Peripheral blood mononuclear cell (PBMCs) routine and fatty acid oxidation‐dependent respiration (a) and oxidative phosphorylation coupling efficiency (b). The complex IV‐linked respiration rate in PBMCs (c). Short‐chain (SC), medium‐chain (MC) and long‐chain (LC) acylcarnitine intracellular content in PBMCs represented as percentage from the rest state content before exercise (d). ROUTINE – respiration in intact cells. LEAK – substrate‐driven respiration before addition of ADP; OXPHOS – oxidative phosphorylation dependent respiration, after addition of ADP. OXPHOS coupling efficiency was calculated as 1‐ Resp.rate at LEAK/Resp.rate at OXPHOS. Values are the mean ± *SEM* from 9 volunteers. *Significantly different from baseline (paired *t* test, *p* < .05)

Moreover, after low‐intensity constant‐load exercise, the ROUTINE respiration was significantly increased by 31% (Figure 4a). Further analysis showed that fatty acid oxidation‐dependent respiration in PBMCs at LEAK (substrate metabolism‐dependent state) was significantly increased by 65%, while respiration rate at oxidative phosphorylation‐dependent (OXPHOS) state the respiration rate was increased by 76% (Figure 4a). These changes resulted in the significantly higher (by 22%) fatty acid oxidation‐dependent OXPHOS coupling efficiency (calculated as coupling control factor as 1‐LEAK respiration/OXPHOS respiration) in PBMCs after low‐intensity constant‐load exercise (Figure 4b).

To evaluate if observed changes in mitochondrial respiration of PBMCs are fatty acid oxidation specific or are related to the overall improvement of mitochondrial electron transfer system capacity, we measured complex IV‐linked respiration at OXPHOS state. As shown in Figure 4c, after low‐intensity constant‐load exercise the complex IV‐linked respiration rate in PBMCs was comparable with respiration rate before exercise, indicating that overall mitochondrial electron transfer system capacity is not changed after exercise. Taken together, these results indicate that low‐intensity constant load‐exercise improves specifically mitochondrial fatty acid oxidation in PBMCs. Acylcarnitine content in PBMC was similar before and after exercise (Figure 4d) indicating that there is no accumulation of acylcarnitines and their synthesis is coupled with oxidation.

**FIGURE 5:**
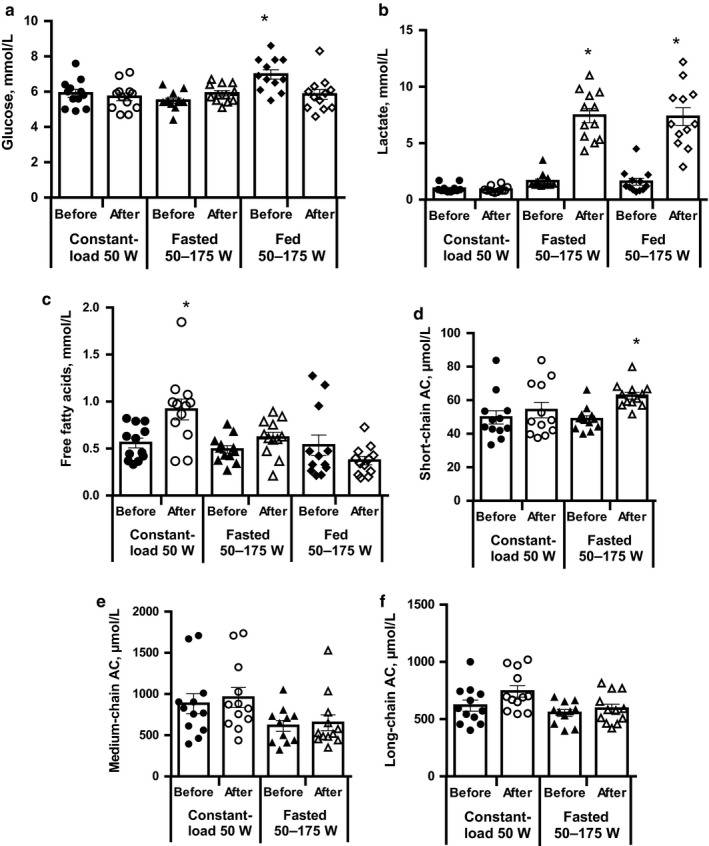
Concentrations of glucose (a), lactate (b), free fatty acids (c) and short‐chain (C2‐C4; d), medium‐chain (C5‐C10; e) and long‐chain (C12‐C18; f) acylcarnitines in plasma before and after exercise. Measurements of plasma levels were performed using commercial reagent sets and the LCMSM method as stated in the Methods section. Values are the mean ± *SEM* from 12 volunteers. *Significantly different from baseline (one‐way repeated measures ANOVA, Tukey's multiple comparisons test, *p* < .05)

The average blood glucose concentration of the fasted study subjects was 5.5 mM, and 30 min after the carbohydrate‐rich meal intake, the glucose concentration significantly increased to up to 7.2 mM (Figure 5a). During the incremental‐load experiment, the blood glucose concentration of the fed subjects decreased to 5.5 mM, while the glucose concentration in the fasted subjects remained unchanged during the whole experiment. The initial blood lactate concentrations in the fed and fasted subjects were similar, ranging from 2.3–3.1 mM (Figure 5b). The measurements of plasma lactate concentration confirmed that all study participants reached the lactate threshold after the incremental‐load experiment but not in the constant‐load experiment (Figure 5b).

The free fatty acid concentration in the fasted subjects was 0.56 ± 0.05 mM. After an incremental‐load exercise in both the fasted and postprandial states, the fatty acid concentrations were 0.62 ± 0.06 and 0.36 ± 0.04 mM, respectively (Figure 5c). After 60 min of low‐intensity constant‐load exercise, the plasma fatty acid concentration significantly increased to 0.92 ± 0.05 mM (Figure 5c), suggesting an at least 2‐fold higher exercise‐induced lipolysis rate in adipocytes. The entire acylcarnitine profile of unsaturated and saturated acylcarnitines from C2‐C18 was measured in plasma samples before and after exercise. The measurements were summarized in three groups: short‐chain (C2‐C4), medium‐chain (C5‐C10), and long‐chain (C12‐C18) acylcarnitines (Figure 5d,e,f). The concentration of short‐chain acylcarnitines before exercise was approximately 48 µM. After constant‐load exercise, the short‐chain acylcarnitine concentration was not significantly changed, while after incremental‐load exercise in fasted subjects, the short‐chain acylcarnitines increased significantly by 33% (Figure 5d). In addition to increased lactate level, this finding suggests diminished mitochondrial oxidative capacity after incremental‐load exercise. Medium‐ and long‐chain acylcarnitines were not significantly changed after either exercise experiment (Figure 5e,f).

## 4 DISCUSSION

The ADA and EASD guidelines state that changes in lifestyle, an exercise, in particular, are an integral part of therapy for type 2 diabetes. It has been shown that exercise not only improves insulin sensitivity and decreases glucose levels in patients with type 2 diabetes and metabolic syndrome (MacLeod et al., 2013; Malin et al., 2013), but also reduces coronary heart disease risk and improves exercise capacity (Golbidi & Laher, 2012; Malin et al., 2013; Sacre et al., 2014). However, exercise intolerance is the primary symptom of various diseases and patients are unable to perform an exercise at high intensities. Therefore in this study, we tested whether the low‐intensity workload is sufficient to induce extra‐muscular effects which could be associated with health benefits. It would be expected that low‐intensity constant‐load exercise would enhance patient adherence because of fewer postexercise adverse effects, such as weakness, muscle pain, and hypoglycaemia, and limit the risk of adverse cardiovascular effects or trauma (O'Keefe et al., 2012).

Chronic inflammation has been added as risk factors for diabetes and cardiovascular diseases as non‐traditional risk factor. Accumulated data have shown that inflammation has a central and inciting role in the development of atherosclerosis leading to increased CVD risk (Ridker, 2018). It has been demonstrated that mitochondrial respiratory dysfunctions of circulating PBMCs could reflect damage to mitochondria due to metabolic or oxidative stress, or the metabolic changes associated with inflammation (Chacko et al., 2014). Thus improving the metabolic adaptation of immune cells would modulate immune cell functions as they are intricately coupled with intracellular metabolism (Weinberg et al., 2015). For instance, HIF‐1α and glycolysis promote proinflammatory activation of macrophages, whereas fatty acid oxidative metabolism regulated by signal transducer and activator of transcription 6 (STAT6) and PPARγ‐coactivator‐1β (PGC‐1β) primes macrophages for the less‐inflammatory alternative M2 state (Vats et al., 2006). Similarly, in CD4(+) Treg cell subsets the promotion of inflammation‐suppressing characteristics require lipid oxidation (Michalek et al., 2011). In this study, low‐intensity exercise increased fatty acid oxidation and improved the efficiency of ATP production from fatty acids in the PBMCs. Thus even low‐intensity exercise acts as an efficient modulator of immune cell metabolism which in the long term could translate to reduced inflammation and prevention of excessive inflammatory responses.

Physical activity is a persistent subject in discussions about the prevention of obesity and health improvement; however, the optimal timing and type of physical activity interventions for untrained individuals remain uncertain. Higher intensity exercise is associated with higher energy expenditure and is expected to correlate with a corresponding increase in fat utilization. However, total energy expenditure during physical activity does not equally represent lipolysis and fat oxidation rates. In our study, healthy individuals with a sedentary lifestyle reached maximal fat oxidation in a cycle ergometery exercise programme at a 50 W workload (36% of VO_2_max). According to our measurements, a heart rate increase of 20–25 BPM corresponds to the optimal whole‐body fat metabolism. At higher workloads, fat oxidation gradually decreased, and at the 150–175 W workloads, the study participants reached exhaustion and declined to continue the cycle ergometry programme. Several studies have reported similarly decreased fat metabolism at high workloads in athletes (Achten & Jeukendrup, 2003; Venables et al., 2005) and overweight volunteers (Bogdanis, Vangelakoudi, & Maridaki, 2008). Thus, the endurance capabilities of untrained individuals greatly limit their abilities to perform vigorous exercise. However, the same participants were able to exercise for 60 min at a 50 W workload and maintain a constant maximal fat oxidation rate. Since the fat oxidation rate was significantly lower at high workloads, in the 60 min low‐intensity exercise study, participants utilized the same amount of total energy and up to 60% more fat compared to those utilized during the incremental workload, thus demonstrating the metabolic benefits of low‐intensity exercise.

The metabolic phenomena characterizing muscle exhaustion include the accumulation of lactate and intermediates of fatty acid metabolism (Xu et al., 2016). During the incremental workload, all participants in this study reached a lactate threshold after 25–30 min and declined the continuation of exercise at a higher intensity workload. Additionally, we observed a significant increase in short‐chain acylcarnitines, suggesting diminished mitochondrial oxidative capacity (Overmyer et al., 2015). In comparison, during 60 min of low‐intensity exercise, increases in plasma lactate and short‐chain acylcarnitine concentrations were not observed. Moreover, in sedentary individuals, muscle ATP production from fatty acid oxidation is highly limited, and the corresponding energy deficiency leads to exhaustion. Thus, the optimal workload is characterized by ATP generation at the optimal fatty acid oxidation rate, and exceeding the maximal fatty acid oxidation capacity leads to the accumulation of fatty acid and carbohydrate metabolism intermediates that inhibit the metabolism of one another and accelerate the mitochondrial energetic crisis. Additionally, exercising in postprandial versus fasted conditions is characterized by different metabolic profiles and impacts on energy balance (Wallis & Gonzalez, 2019). Similar to the results of previous studies (Vieira, Costa, Macedo, Coconcelli, & Kruel, 2016), we observed that aerobic exercise performed in the fasted state induces greater fat oxidation. This finding leads to the conclusion that a fasted state is the preferred state for fat burning during low‐intensity exercise. At high workloads, the difference in the fat oxidation rate becomes less pronounced because carbohydrate oxidation is dominant over fat oxidation.

The plasma free fatty acid concentration is defined by the lipolysis rate in adipocytes and the uptake by muscle and other tissue (Frayn, 2002). The rates of lipolysis and efflux of fatty acids from adipocytes in the fasted state are significantly higher than those in the postprandial state (Frayn, 2002). In this study, only after constant‐load exercise, the plasma fatty acid concentration increased significantly. Since both the fatty acid utilization rate and the plasma concentrations of free fatty acids after low‐intensity exercise were 1.5 times higher, prolonged low‐intensity exercise‐induced more than a 2‐fold higher lipolysis rate in adipocytes than vigorous‐intensity exercise.

The maximal fat oxidation rate for study participants with a sedentary lifestyle was 17 g/hr (153 kcal/hr) or 0.22 g h^−1^ kg^−1^. Therefore, constantly exercising for 300 min/week would result in approximately 4.2 kg/year of utilized fat. Additional benefits are usually expected from the exercise‐stimulated metabolism of carbohydrates. However, only fat oxidation has a primary effect on lipid stores, while carbohydrate utilization would be beneficial to prevent excess carbohydrates from being converted into fat. Therefore, subjects with balanced or even reduced‐calorie diets would not gain any additional weight due to increased carbohydrate utilization. In countless trials, effective initial weight loss was followed by partial or complete weight regain (Byrne & Hills, 2018; Maclean, Bergouignan, Cornier, & Jackman, 2011; Wadden, Webb, Moran, & Bailer, 2012). To maintain weight loss, individuals must adhere to lifestyle changes that counteract physiological adaptations favoring weight regain (Greenway, 2015; Maclean et al., 2011; Montesi et al., 2016). In addition to a low‐calorie diet, physical activity has a significant impact during the weight‐loss period and becomes essential for weight maintenance (Curioni & Lourenco, 2005; Unick et al., 2017; Wu, Gao, Chen, & van Dam, 2009). In a long‐term period, greater motivation and adherence is expected for low‐intensity activities such as everyday walking and cycling or leisure activities such as dancing (Mangeri, Montesi, Forlani, Dalle Grave, & Marchesini, 2014; Middleton, Anton, & Perri, 2013; Perri et al., 2002).

In conclusion, for the first time was demonstrated that low‐intensity exercise improves bioenergetics and increases fat oxidation in PBMCs and thus acts as an efficient modulator of immune cell metabolism which might translate to reduced inflammation. During 60 min of low‐intensity exercise, a higher lipolysis rate was observed, 30% more of total energy was utilized and two times more fat was oxidized than during the incremental‐load exercise workout. Vigorous‐intensity exercise is duration‐limited because it leads to rapid exhaustion, a dramatic increase in heart rate and increased cardiovascular risks.

## CONFLICT OF INTEREST

There is no conflict of interest.

## Data Availability

The datasets generated and analyzed during the current study are available from the corresponding author on reasonable request.
